# TSInsight: A Local-Global Attribution Framework for Interpretability in Time Series Data

**DOI:** 10.3390/s21217373

**Published:** 2021-11-05

**Authors:** Shoaib Ahmed Siddiqui, Dominique Mercier, Andreas Dengel, Sheraz Ahmed

**Affiliations:** 1 German Research Center for Artificial Intelligence (DFKI), 67663 Kaiserslautern, Germany; andreas.dengel@dfki.de (A.D.); sheraz.ahmed@dfki.de (S.A.); 2 Department of Computer Science, TU Kaiserslautern, 67663 Kaiserslautern, Germany

**Keywords:** interpretability, time series analysis, feature attribution, deep learning, auto-encoder, feature importance, demystification

## Abstract

With the rise in the employment of deep learning methods in safety-critical scenarios, interpretability is more essential than ever before. Although many different directions regarding interpretability have been explored for visual modalities, time series data has been neglected, with only a handful of methods tested due to their poor intelligibility. We approach the problem of interpretability in a novel way by proposing TSInsight, where we attach an auto-encoder to the classifier with a sparsity-inducing norm on its output and fine-tune it based on the gradients from the classifier and a reconstruction penalty. TSInsight learns to preserve features that are important for prediction by the classifier and suppresses those that are irrelevant, i.e., serves as a feature attribution method to boost the interpretability. In contrast to most other attribution frameworks, TSInsight is capable of generating both instance-based and model-based explanations. We evaluated TSInsight along with nine other commonly used attribution methods on eight different time series datasets to validate its efficacy. The evaluation results show that TSInsight naturally achieves output space contraction; therefore, it is an effective tool for the interpretability of deep time series models.

## 1. Introduction

Deep learning models have been at the forefront of technology in a range of different domains including image classification [1], object detection [2], speech recognition [3], text recognition [4] and image captioning [5]. These models are particularly effective in automatically discovering useful features. However, this automatic feature extraction comes at the cost of a lack of transparency of the system. Therefore, despite these advances, their employment in safety-critical domains, such as finance [6], self-driving cars [7] and medicine [8], is limited due to the lack of interpretability of the decision made by the network.

Numerous efforts have been made for the interpretation of these black-box models. These efforts can mainly be classified into two separate directions. The first set of strategies focuses on making the network itself interpretable by trading off some performance. The second set of strategies focuses on explaining a pretrained model, i.e., they try to infer the reason for a particular prediction. However, all of these methods have been particularly developed and tested for visual modalities which are directly intelligible for humans. Transferring methodologies developed for visual modalities to time series data is difficult due to the non-intuitive nature of time series. Therefore, only a handful of methods have been focused on explaining time series models in the past [9,10].

We approach the attribution problem in a novel way by attaching an auto-encoder on top of the classifier. The auto-encoder is fine-tuned based on the gradients from the classifier. Rather than optimizing the auto-encoder to reconstruct the whole input, we optimize the network to only reconstruct parts which are useful for the classifier, i.e., are correlated or causal for the prediction. Although auto-encoders have been used to understand the classifier in the past [11], we extend this to the task of attribution, where relevant features of the input have to be highlighted. For this purpose, it is important for the reconstructions to be aligned with the inputs. Furthermore, to serve as attribution, the model should only reconstruct parts which are correlated or causal for the prediction. TSInsight solves these problems with a sparsity-inducing norm as well as a reconstruction penalty. In particular, the contributions of this paper are twofold:

- A novel attribution method for time series data which makes it much easier to interpret the decision of any deep learning model. The method also leverages dataset-level insights when explaining individual decisions in contrast to other attribution methods.
- A detailed analysis of the information captured by 11 different attribution techniques using the suppression test on 8 different time series datasets, setting up a strong benchmark.

The rest of the paper is structured as follows. Section 2 covers most of the prominent prior work in the area of interpretability of deep learning models. Section 3 provides an in-depth view of TSInsight. Section 4.2 provides details regarding the datasets we employed in our experimentation. Section 5 provides an overview of the main results. Section 5.1 analyzes the out-of-the-box properties achieved by TSInsight. Finally, the paper is concluded with the concluding remarks in Section 6.

## 2. Related Work

Since the resurgence of deep learning in 2012 after a deep network comprehensively outperformed its feature-engineered counterparts [1] on the ImageNet visual recognition challenge comprising of 1.2 million images [12], deep learning has been integrated into a range of different applications to gain unprecedented levels of improvement. Significant efforts have been made in the past regarding the interpretability of deep models, specifically for image modality. These methods are mainly categorized into two different streams where the first stream is focused on explaining the decisions of a pretrained network while the second stream is directed towards making models more interpretable by trading off accuracy.

The first stream for explainable systems, which attempts to explain pretrained models using attribution techniques, has been a major focus of research in the few past years. The most common strategy is to visualize the filters of the deep model [11,13,14,15,16]. This is very effective for visual modalities since images are directly intelligible for humans. Zeiler and Fergus [13] introduced a deconvnet layer to understand the intermediate representations of the network. They not only visualized the network, but were also able to improve the network based on these visualizations to achieve state-of-the-art performance on ImageNet [12]. Simonyan et al. [14] proposed a method to visualize class-specific saliency maps. Yosinski et al. [15] developed a visualization framework for image-based deep learning models. They tried to visualize the features that a particular filter was responding to by using regularized optimization. Instead of using first-order gradients, [16] introduced a layer-wise relevance propagation (LRP) framework which identified the relevant portions of the image by distributing the contribution to the incoming nodes. Smilkov et al. [17] introduced the SmoothGrad method where they computed the mean gradients after adding small random noise sampled from a zero-mean Gaussian distribution to the original point. Sundararajan et al. [18] introduced the integrated gradients method, which works by computing the average gradient from the original point to the baseline input (zero-image in their case) at regular intervals. Guo et al. [19] used a Bayesian non-parametric regression mixture model with multiple elastic nets to extract generalizable insights from the trained model. Recently, Fong et al. [20] presented the extremal perturbation method where they solve an optimization problem to discover the minimum enclosing mask for an image that retains the network’s predictive performance. Either these methods are not directly applicable to time series data, or are inferior in terms of intelligibility for time series data.

Palacio et al. [11] introduced yet another approach to understand a deep model by leveraging auto-encoders. After training both the classifier and the auto-encoder in isolation, they attached the auto-encoder to the head of the classifier and fine-tuned only the decoder, freezing the parameters of the classifier and the encoder. This transforms the decoder to focus on features which are relevant for the network. Applying this method directly to time series yields no interesting insights (Figure 2c) into the network’s preference for input. Therefore, this method is strictly a special case of the TSInsight’s formulation.

In the second stream for explainable systems, [21] proposed self-explaining neural networks (SENN) where they learn two different networks. The first network is the concept encoder which encodes different concepts, while the second network learns the weightings of these concepts. This transforms the system into a linear problem with a set of features making it easily interpretable for humans. SENN trade off accuracy in favor of interpretability. Kim et al. [7] attached a second network (video-to-text) to the classifier which was responsible for the production of natural language-based explanation of the decisions taken by the network using the saliency information from the classifier. This framework relies on long short-term memory (LSTM) for the generation of the descriptions, adding yet another level of opaqueness making it hard to decipher whether the error originated from the classification network or from the explanation generator.

Kumar et al. [9] made the first attempt to understand deep learning models for time series analysis where they specifically focused on financial data. They computed the input saliency based on the first-order gradients of the network. Siddiqui et al. [10] proposed an influence computation framework which enabled exploration of the network at the filter level by computing the per filter saliency map and filter importance, again based on first-order gradients. However, both methods lack in providing useful insights due to the noise inherent to first-order gradients. Another major limitation of saliency-based methods is the sole use of local information. Therefore, TSInsight significantly supersedes in the identification of the important regions of the input using a combination of both local information for that particular example along with generalizable insights extracted from the entire dataset in order to reach a particular description.

Due to the use of auto-encoders, TSInsight is inherently related to sparse [22] and contractive auto-encoders [23]. In sparse auto-encoders [22], the sparsity is induced on the hidden representation by minimizing the KL-divergence between the average activations and a hyperparameter which defines the fraction of non-zero units. This KL-divergence is a necessity for sigmoid-based activation functions. However, in our case, the sparsity is induced directly on the output of the auto-encoder, which introduces a contraction on the input space of the classifier, and can directly be achieved by using the Manhattan norm on the activations as we obtain real-valued outputs. Albeit sparsity being introduced in both cases, the sparsity in the case of sparse auto-encoders is not useful for interpretability. In the case of contractive auto-encoders [23], contraction mapping is introduced by penalizing the Frobenius norm of the Jacobian of the encoder along with the reconstruction error. This makes the learned representation invariant to minor perturbations in the input. TSInsight, on the other hand, induces a contraction on the input space for interpretability, thus, favoring sparsity inducing norm.

Ghaderpour et al. [24] reviewed the change detection and general time series analysis literature. We refer readers to the recent survey from Rojat et al. [25] for a more in-depth overview of the existing time series interpretability methods covering different perspectives relevant for interpretability, existing methods and challenges. Their work covers ante-hoc and post-hoc methods. However, in this work, we focus on post-hoc instance-based analysis.

## 3. Method

**Figure 1:**
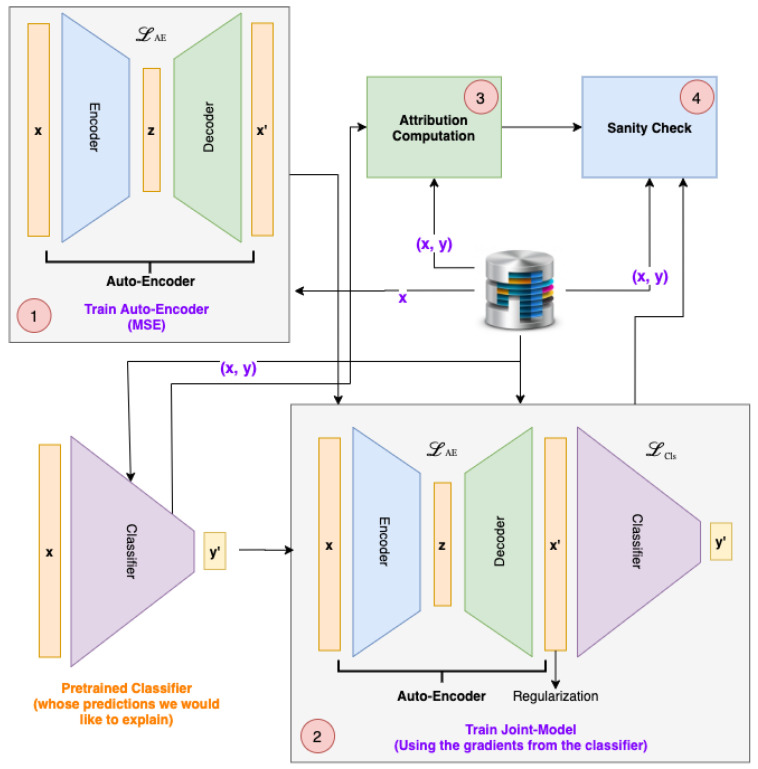
System pipeline.

The overview of our methodology is presented in Figure 1. As the purpose of TSInsight is to explain the predictions of a pretrained model, we train a vanilla auto-encoder on the desired dataset as the first step (indicated as step 1 in the figure). Once the auto-encoder is trained, we stack the auto-encoder on top of the pretrained classifier to obtain a combined model. We then only fine-tune the auto-encoder within the combined model using the gradients from the classifier, using a specific loss function to highlight the causal/correlated points (indicated as step 2 in the figure). Finally, we compute the attributions from the trained auto-encoder (step 3) followed by the sanity check using our suppression test (step 4). We will first cover some basic background and then dive into the formulation of the problem presented by Palacio et al. [11]. We will then present the proposed formulation, adapting the basic one for the interpretability of deep learning-based time series models.

### 3.1. Pretrained Classifier

A classifier (Φ:X↦Y) is a map from the input space X to the output space Y. As the emphasis of TSInsight is interpretability, we assume the presence of a pretrained classifier whose predictions we are willing to explain. For this purpose, we trained a classifier using standard regularized risk minimization on the given dataset. The objective can be written as:

(1) $$W^{*}=\underset{W}{\arg\;\min}\;\frac{1}{\left|X\right|}\sum_{(x,y)\in X\times Y}L\left(\Phi (x;W^{*}),y\right)+\lambda {\left||W|\right|}_{2}^{2}$$

where Φ defines the mapping from the input space X to the output space Y, while L corresponds to the classification loss (assumed to be cross-entropy in our case). Furthermore, ${\left||.|\right|}_{p}$ represents the $L_{p}$ norm. Specific instances of $L_{p}$ norm that we use within this paper are $L_{1}$ and $L_{2}$ norm. The $L_{1}$ norm is computed by summing up the absolute values of the given vector (${\left||x|\right|}_{1}=\sum_{i=1}^{d}\left|x_{i}\right|$). Similarly, the $L_{2}$ norm is computed by taking the square root of the sum of squared values of the given vector (${\left||x|\right|}_{2}=\sqrt{\sum_{i=1}^{d}x_{i}^{2}}$). The objective also includes a regularization term ${\left||W|\right|}_{2}^{2}$ with an associated hyperparameter λ to define the relative importance of the classification objective and the simplicity of the hypothesis class. $W^{*}$ denotes the final set of parameters obtained after optimization.

### 3.2. Auto-Encoder

**Figure 2:**
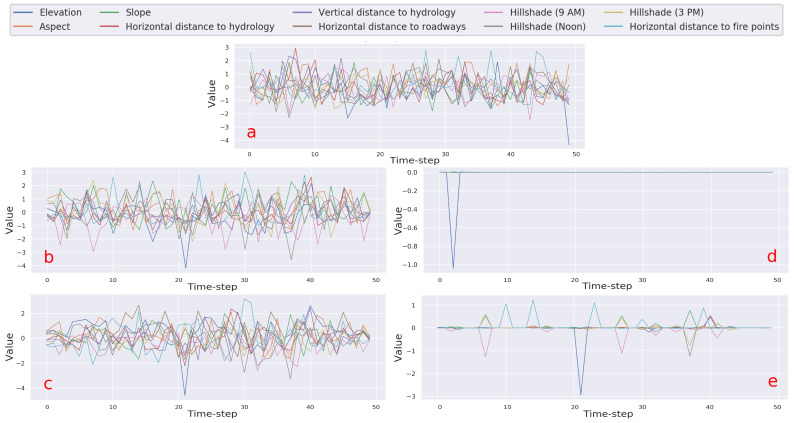
Comparison of different auto-encoder outputs (**a**) original input, (**b**) reconstruction from the vanilla auto-encoder, (**c**) Palacio et al. [11], (**d**) auto-encoder fine-tuned with sparsity and (**e**) TSInsight.

An auto-encoder (D∘E:X↦X) is a neural network where the defined objective is to reconstruct the provided input by embedding it into an arbitrary feature space F, therefore, it is a mapping from the input space X to the input space itself X after passing it through the feature space F. The auto-encoder is usually trained through mean-squared error as the loss function. The optimization problem for an auto-encoder is:

(2) $$\begin{array}{c} \left(W_{E}^{*},W_{D}^{*}\right)=\underset{W_{E},W_{D}}{\arg\;\min}\;\frac{1}{\left|X\right|}\sum_{x\in X}{\left||x-D\left(E\left(x;W_{E}\right);W_{D}\right)|\right|}_{2}^{2}+\lambda \left({\left||W_{E}|\right|}_{2}^{2}+{\left||W_{D}|\right|}_{2}^{2}\right) \end{array}$$

where *E* defines the encoder with parameters $W_{E}$ while *D* defines the decoder with parameters $W_{D}$. Similar to the case of classifier, we train the auto-encoder using regularized risk minimization on a particular dataset. A sample reconstruction from the auto-encoder is visualized in Figure 2b for the forest cover dataset. It can be seen that the network was able to reconstruct the input.

### 3.3. Formulation by Palacio et al.

Palacio et al. (2018) [11] presented an approach for discovering the preference the network had for the input by attaching the auto-encoder on top of the classifier. The auto-encoder was fine-tuned using the gradients from the classifier. The new optimization problem for fine-tuning the auto-encoder is:

(3) $$\begin{array}{c} \left(W_{E}^{{}^{\prime }},W_{D}^{{}^{\prime }}\right)=\underset{W_{E}^{*},W_{D}^{*}}{\arg\;\min}\;\frac{1}{\left|X\right|}\sum_{(x,y)\in X\times Y}L\left(\Phi \left(D\left(E\left(x;W_{E}^{*}\right);W_{D}^{*}\right);W^{*}\right),y\right)+\\ \lambda \left({\left||W_{E}^{*}|\right|}_{2}^{2}+{\left||W_{D}^{*}|\right|}_{2}^{2}\right) \end{array}$$

where $W_{E}^{*}$ and $W_{D}^{*}$ are initialized from the auto-encoder weights obtained after solving the optimization problem specified in Equation (2), while $W^{*}$ is obtained by solving the optimization problem specified in Equation (1). This formulation is slightly different from the one proposed by Palacio et al. (2018) where they only fine-tuned the decoder part of the auto-encoder, while we update both the encoder as well as the decoder as it is a much more natural formulation as compared to only fine-tuning the decoder. This complete fine-tuning is significantly more important once we move towards advanced formulations since we would like the network to also adapt the encoding in order to better focus on important features. Fine-tuning only the decoder will only change the output without the network learning to compress the signal itself.

### 3.4. TSInsight: The Proposed Formulation

In contrast to the findings of Palacio et al. (2018) [11] for the image domain, directly optimizing the objective defined in Equation (3) for time series yields no interesting insights into the input preferred by the network. This effect is amplified with an increase in dataset complexity. Figure 2c presents an example from the forest cover dataset. Even though the network was able to reconstruct the anomaly present in the dataset, this resulted in a loss of spatial information. Since attribution maps highlight the regions in the input space which are relevant for the prediction, this highlights the limitation of the original scheme to be used as an attribution method. Therefore, instead of optimizing this raw objective, we modify the objective by adding the sparsity-inducing norm on the output of the auto-encoder. Inducing sparsity on the auto-encoder’s output forces the network to only reproduce relevant regions of the input to the classifier since the auto-encoder is optimized using the gradients from the classifier. The use of this sparsity-inducing norm stems from our motivation to obtain the most sparse attribution that retains the prediction. A trivial solution for obtaining attributions is just to predict the whole sequence to be causal/correlated with the prediction if that would have not been the case. Albeit being correct, the attribution obtained in this case would not be useful. Therefore, for human understanding, attributing the prediction to the smallest region possible is important. This has been termed as the complexity of the explanation in the past [26], and our sparsity-based framework focuses on finding the explanation with the least complexity.

However, just optimizing for sparsity introduces a misalignment between the reconstruction and the input, as visualized in Figure 2d. In order to ensure alignment between the two sequences, we additionally introduce a reconstruction loss into the final objective. Therefore, the proposed TSInsight optimization objective is:

(4) $$\begin{array}{c} \left(W_{E}^{{}^{\prime }},W_{D}^{{}^{\prime }}\right)=\underset{W_{E}^{*},W_{D}^{*}}{\arg\;\min}\;\frac{1}{\left|X\right|}\sum_{(x,y)\in X\times Y}[L\left(\Phi \left(D\left(E\left(x;W_{E}^{*}\right);W_{D}^{*}\right);W^{*}\right),y\right)+\\ \gamma \left({\left||x-D\left(E\left(x;W_{E}^{*}\right);W_{D}^{*}\right)|\right|}_{2}^{2}\right)+\beta \left({\left||D\left(E\left(x;W_{E}^{*}\right);W_{D}^{*}\right)|\right|}_{1}\right)]+\lambda \left({\left||W_{E}^{*}|\right|}_{2}^{2}+{\left||W_{D}^{*}|\right|}_{2}^{2}\right) \end{array}$$

where L represents the classification loss function, which is cross-entropy in our case, Φ denotes the classifier with pretrained weights $W^{*}$, while *E* and *D* denotes the encoder and decoder, respectively, with the corresponding pretrained weights $W_{E}^{*}$ and $W_{D}^{*}$. We introduce two new hyperparameters, γ and β. γ controls the auto-encoder’s focus on reconstruction of the input. β, on the other hand, controls the sparsity enforced on the output of the auto-encoder. After training the auto-encoder with the TSInsight objective function, the output is both sparse as well as aligned with the input, as evident from Figure 2e.

The aforementioned hyperparameters play an essential role for TSInsight to provide useful insights into the model’s behavior. Performing a grid search to determine this value is not possible as the large values of β result in models which are more interpretable but inferior in terms of performance, therefore, presenting a trade-off between performance and interpretability, which is difficult to quantify. Although we found the manual tuning of hyperparameters to be superior, we also investigated the employment of feature importance measures [10,27] for the automated selection of these hyperparameters (β and γ). The simplest candidate for this importance measure is saliency:

(5) $$I\left(x\right)=\frac{\partial a^{L}}{\partial x}$$

where *L* denotes the number of layers in the classifier and $a^{L}$ denotes the activations of the last layer in the classifier. This saliency-based importance computation is only based on the classifier. Once the feature importance values are computed, they are scaled in the range of (0, 1), as shown in Equation (6), to serve as the corresponding reconstruction weight, i.e., γ (Equation (7)). The inverted importance values then serve as the corresponding sparsity weight, i.e., β, as highlighted in Equation (8).

(6) $$I\left(x\right)=\frac{I\left(x\right)-\min_{j}I{\left(x\right)}_{j}}{\max_{j}I{\left(x\right)}_{j}-\min_{j}I{\left(x\right)}_{j}}$$

(7) $$\gamma^{*}\left(x\right)=I\left(x\right)$$

(8) $$\beta^{*}\left(x\right)=1.0-I\left(x\right)$$

Therefore, the objective imposing sparsity on the classifier can be written as:

(9) $$\begin{array}{c} \gamma \left({\left||x-D\left(E\left(x;W_{E}^{*}\right);W_{D}^{*}\right)|\right|}_{2}^{2}\right)+\beta \left({\left||D\left(E\left(x;W_{E}^{*}\right);W_{D}^{*}\right)|\right|}_{1}\right)\Rightarrow \\ \;C\times {\left||D\left(E\left(x;W_{E}^{*}\right);W_{D}^{*}\right)⊙\beta^{*}\left(x\right)|\right|}_{1}+{\left||\left(x-D\left(E\left(x;W_{E}^{*}\right);W_{D}^{*}\right)\right)⊙\gamma^{*}\left(x\right)|\right|}_{2}^{2} \end{array}$$

where ⊙ corresponds to the Hadamard (element-wise) product evading the need to manually tune the hyperparameters (β and γ). In contrast to the instance-based value of β, we used the average saliency value in our experiments. This ensures that the activations are not sufficiently penalized so as to significantly impact the performance of the classifier. Due to the low relative magnitude of the sparsity term, we scaled it by a constant factor *C*. Although a new hyperparameter *C* has been introduced instead of the two old hyperparameters (β and γ), the value of *C* can be easily fixed based on the relative contribution of the two terms. We used C=10 in all of our experiments.

## 4. Experimental Setup

This section will cover the evaluation setup that we used to establish the utility of TSInsight in comparison to other commonly used attribution techniques. We will first define the evaluation metric we used to compare different attribution techniques. Then, we will discuss the 8 different datasets that we used in our experimental study, followed by the 11 different attribution techniques that we compared.

### 4.1. Evaluation Metric

A commonly used metric to compare model attributions in visual modalities is via the pointing game or suppression test [20]. Since the pointing game is not directly applicable to time series data, we compare TSInsight with other attribution techniques using the suppression test. The suppression test attempts to quantify the quality of the attribution by just preserving parts of the input that are considered to be important by the method. This suppressed input is then passed to the classifier. If the selected points are indeed causal/correlated to the prediction generated by the classifier, no evident effect on the prediction should be observed. On the other hand, if the points highlighted by the attribution technique are not the most important ones for prediction, the network’s prediction will change. It is important to note that unless there is a high amount of sparsity already present in the original signal, suppressing the signal itself will result in a loss of accuracy for the classifier since there is a slight mismatch for the classifier for the inputs seen during training. We compared TSInsight with a range of different saliency methods.

### 4.2. Datasets

We employed 8 different time series datasets in our study. The summary of these datasets is available in Table 1. We will now cover each of these datasets in detail. Besides the synthetic anomaly detection dataset, all of the datasets were taken from the University of East Anglia (UEA) Digital Repository [28].

**Synthetic Anomaly Detection Dataset:** The synthetic anomaly detection dataset [10] is a synthetic dataset comprising three different channels referring to the pressure, temperature and torque values of a machine running in a production setting where the task is to detect anomalies. The dataset only contains point anomalies. If a point anomaly is present in a sequence, the whole sequence is marked as anomalous. Anomalies were intentionally never introduced on the pressure signal in order to identify the treatment of the network to that particular channel.

**Electric Devices Dataset:** The electric devices dataset [29] is a small subset of the data collected as part of the UK government’s sponsored study, *Powering the Nation*. The aim of this study was to reduce the UK’s carbon footprint. The electric devices dataset is comprised of data from 251 households, sampled in two-minute intervals over a month.

**Character Trajectories Dataset:** The character trajectories dataset contains handwritten characters using a Wacom tablet. Only three dimensions are kept for the final dataset, which includes x, y and pen tip force. The sampling rate was set to be 200 Hz. The data was numerically differentiated and Gaussian smoothened with σ=2. The task is to classify the characters into 20 different classes.

**FordA Dataset:** The FordA dataset was originally used for a competition organized by the Institute of Electrical and Electronics Engineers (IEEE) in the IEEE World Congress on Computational Intelligence (2008). It is a binary classification problem where the task is to identify whether a certain symptom exists in the automotive subsystem. FordA dataset was collected with minimal noise contamination in typical operating conditions.

**Forest Cover Dataset:** The forest cover dataset [30] has been adapted from the University of California, Irvine (UCI) Machine Learning Repository for the classification of forest cover type from cartographic variables. The dataset has been transformed into an anomaly detection dataset by selecting only 10 quantitative attributes out of a total of 54. Instances from the second class were considered to be normal, while instances from the fourth class were considered to be anomalous. The ratio of the anomalies to normal data points is 0.9%. Since only two classes were considered, the rest of them were discarded.

**WESAD Dataset:** The WESAD dataset [31] is a classification dataset introduced by Bosch for a person’s affective state classification with three different classes, namely, neutral, amusement and stress.

**ECG Thorax Dataset:** The non-invasive fetal ECG Thorax dataset is a classification dataset comprising of 42 classes.

**UWave Gesture Dataset:** The wave gesture dataset [32] contains accelerometer data where the task is to recognize 8 different gestures.

### 4.3. Attribution Techniques

We compared TSInsight against a range a commonly employed attribution techniques. Each attribution method provided us with an estimate of the features’ importance which we used to suppress the signal. In all of the cases, we used the absolute magnitude of the corresponding feature attribution method to preserve the most important input features. When using ReLU networks with batch norm (zero baseline and no bias term), ϵ−LRP and DeepLift were shown to be similar to input⊙gradient [33], therefore, we compare only against input⊙gradient as we satisfy both these conditions. We do not compute class-specific saliency, but instead compute the saliency w.r.t. all the output classes. For all of the methods computing class-specific activations maps, e.g., GradCAM, guided GradCAM and occlusion sensitivity, we used the class with the maximum predicted score as our target. The description of the 11 different attribution techniques evaluated in this study is provided below:

**None:** None refers to the absence of any importance measure. Therefore, in this case, the complete input is passed on to the classifier without any suppression for comparison.

**Random:** Random points from the input are suppressed in this case.

**Input Magnitude:** We treat the absolute magnitude of the input to be a proxy for the features’ importance.

**Occlusion sensitivity:** We iterate over different input channels and positions and mask the corresponding input features with a filter size of 3 and compute the difference in the confidence score of the predicted class (i.e., the class with the maximum score on the original input). We treat this sensitivity score as the features’ importance. This is a brute-force measure of feature importance and employed commonly in prior literature as it served as a strong baseline in our experiments [13]. A major limitation of occlusion sensitivity is its execution speed since it requires iterating over the complete input running inference numerous times.

**TSInsight:** We treat the absolute magnitude of the output from the auto-encoder of TSInsight as features’ importance.

**Palacio et al.:** Similar to TSInsight, we use the absolute magnitude of the auto-encoder’s output as the features’ importance [11].

**Gradient:** We use the absolute value of the raw gradient of the classifier w.r.t. to all of the classes as the features’ importance [9,10].

**Gradient**⊙**Input:** We compute the Hadamard (element-wise) product between the gradient and the input, and use its absolute magnitude as the features’ importance [18].

**Integrated Gradients:** We use absolute value of the integrated gradient with 100 discrete steps between the input and the baseline (which was zero in our case) as the features’ importance [18].

**SmoothGrad:** We use the absolute value of the smoothened gradient computed by using 100 different random noise vectors sampled from a Gaussian distribution with zero mean, and a variance of $2/(max_{j}x_{j}-min_{j}x_{j})$ where x was the input as the features’ importance measure [17].

**Guided Backpropagation:** We use the absolute value of the gradient provided by guided backpropagation [34]. In this case, all the ReLU layers were replaced with guided ReLU layers which masks negative gradients, hence filtering out negative influences for a particular class to improve the visualization.

**GradCAM:** We use the absolute value of gradient-based class activation map (GradCAM) [35] as our feature importance measure. GradCAM computes the importance of the different filters present in the input in order to come up with a metric to score the overall output. Since GradCAM visualizes a class activation map, we used the predicted class as the target for visualization.

**Guided GradCAM:** Guided GradCAM [35] is a guided variant of GradCAM which performs a Hadamard product (point-wise) of the signal from guided backpropagation and GradCAM to obtain a guided GradCAM. We again use the absolute value of the guided GradCAM output as importance measure.

### 4.4. Hyperparameters

The hyperparameters used for training our models are presented in Table 2. These hyperparameters are associated with general model training, except for the two hyperparameters introduced by TSInsight (γ and β). The impact of these method-specific hyperparameters is shown in Table 3.

## 5. Results

The results we obtained with the proposed formulation were highly intelligible for the datasets we employed in this study. TSInsight produced a sparse representation of the input focusing only on the salient regions. In addition to interpretability, with careful tuning of the hyperparameters, TSInsight outperformed the pretrained classifier in terms of accuracy for most of the cases, which is evident from Table 3. However, it is important to note that TSInsight is not designed for the purpose of performance, but rather for interpretability. Therefore, we expect that the performance will drop in many cases depending on the amount of sparsity enforced.

**Figure 3:**
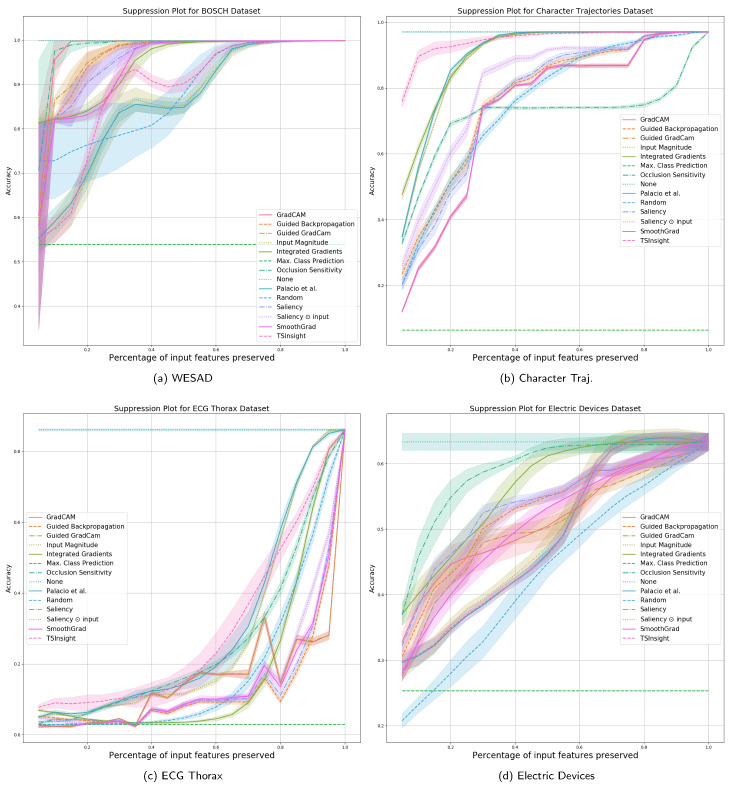
Suppression results (1/2) against a large number of baseline methods computed using 5 random runs (best viewed digitally).

**Figure 4:**
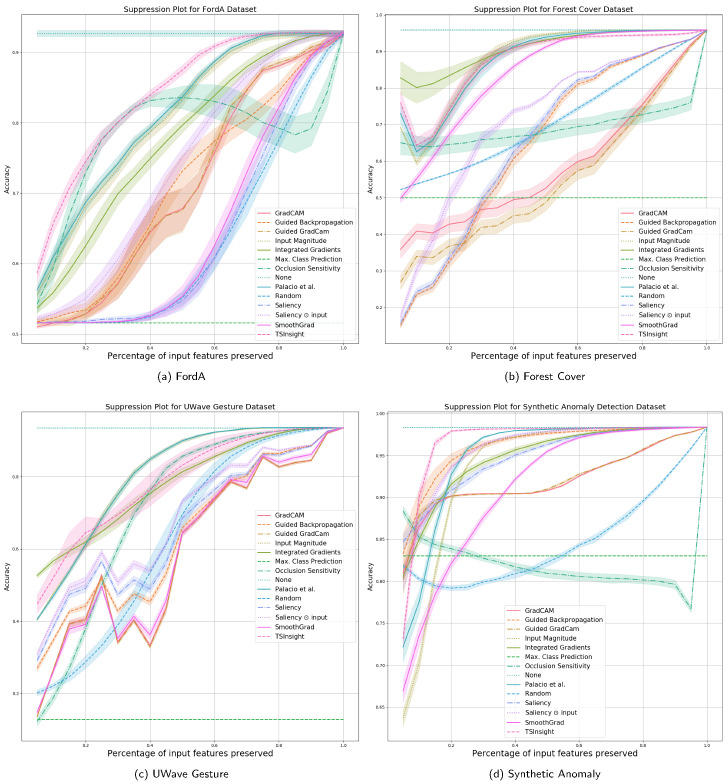
Suppression results (2/2) against a large number of baseline methods computed using 5 random runs (best viewed digitally).

As described in Section 4.1, we compare the performance of different attribution techniques using the input suppression test. Since the input suppression test attempts to suppress the input signal, which is not deemed to be important by the attribution, a good attribution method should result in a negligible loss in performance when suppressing the input, specifically when considering a small number of input points to be suppressed. The results with a different amount of suppression are visualized in Figure 3 and Figure 4, which are computed based on five random runs.

Since the datasets were picked to maximize diversity in terms of the features, there is no single method which can be perfectly generalized to all the datasets. It is evident from the figure that TSInsight significantly superseded the other methods on four out of eight of the datasets, which includes character trajectories, ECG thorax, FordA and synthetic anomaly detection dataset. Occlusion sensitivity served as one of the strongest baselines throughout the different datasets as it directly captures the influence of the feature by explicitly masking the input, which is itself quite similar to the suppression test. It is interesting to note that in cases where TSInsight was not able to retain high accuracy after suppression, almost all of the pure gradient-based methods struggled in those cases. As guided backpropagation overrides the backpropagation phase, it is not considered as a pure gradient-based method [33], which makes it superior in terms of performance as compared to other methods when the gradient is misleading. It is also interesting to note that for the WESAD dataset, none of the most competing methods were in the top list due to the extremely different nature of the dataset. TSInsight turned out to be the most competitive saliency estimator on average in comparison to all of the other attribution techniques tested.

**Figure 5:**
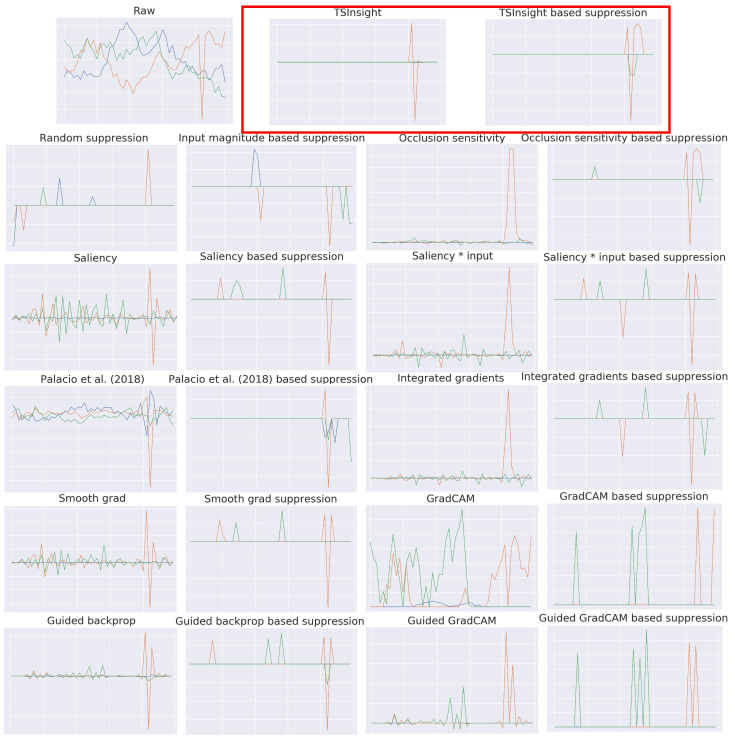
Output from different attribution methods as well as the input after suppressing all the points except the top 5% highlighted by the corresponding attribution method on an anomalous example from the synthetic anomaly detection dataset (best viewed digitally). All methods were able to correctly identify the anomalous spike given the simplicity of this dataset. However, qualitative differences exist between different methods.

In order to qualitatively assess the attribution provided by TSInsight, we visualize an anomalous example from the synthetic anomaly detection dataset in Figure 5 along with the attributions from all of the commonly employed attribution techniques (listed in Section 4.3). Since there were only a few relevant discriminative points in the case of forest cover and synthetic anomaly detection datasets, TSInsight suppressed most of the input, making the decision directly interpretable. This highlights the fact that alongside the numbers, TSInsight was also able to produce the most plausible explanations.

### 5.1. Properties of TSInsight

We will now discuss some of the interesting properties that TSInsight achieves out-of-the-box, which includes output space contraction, its generic applicability and model-based (global) explanations. Since TSInsight induces a contraction in the input space, this also results in slight gains in terms of adversarial robustness. However, these gains are not consistent over many datasets and strong adversaries, therefore, they are omitted for clarity here. An in-depth evaluation of the adversarial robustness of TSInsight can be an interesting future direction.

#### 5.1.1. Model-Based vs. Instance-Based Explanations

Since TSInsight poses the attribution problem itself as an optimization objective, the data based on which this optimization problem is solved defines the explanation scope. If the optimization problem is solved for the complete dataset, this tunes the auto-encoder to be a generic feature extractor, enabling extraction of model/dataset-level insights using the attribution. In contrary, if the optimization problem is solved for a particular input, the auto-encoder discovers an instance’s attribution. This is contrary to most other attribution techniques which are only instance specific.

#### 5.1.2. Auto-Encoder’s Jacobian Spectrum Analysis

**Figure 6:**
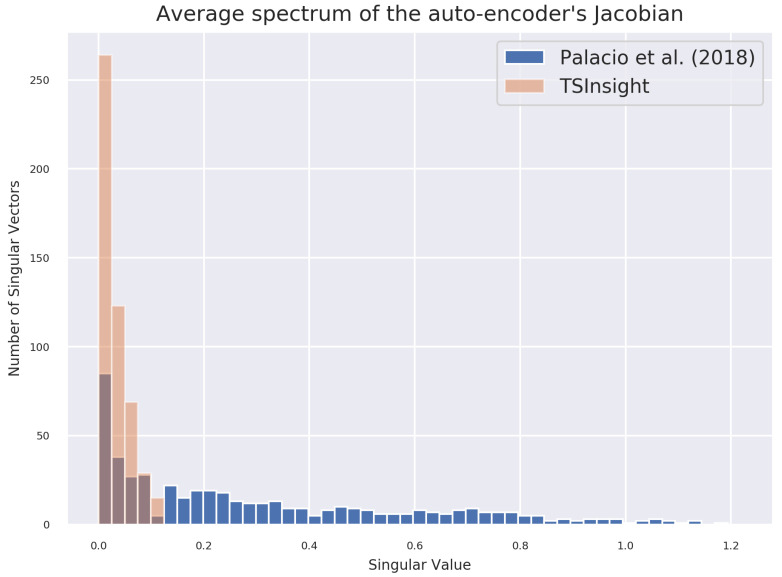
Spectrum analysis of the auto-encoder’s average Jacobian computed over the entire test set of the forest cover dataset. The sharp decrease in the spectrum for TSInsight suggests that the network was successful in inducing a contraction of the input space.

Figure 6 visualizes the histogram of singular values of the average Jacobian on the test set of the forest cover dataset. We compare the spectrum of the formulation from [11] and TSInsight. It is evident from the figure that most of the singular values for TSInsight are close to zero, indicating a contraction being induced in those directions. This is similar to the contraction induced in contractive auto-encoders [23] without explicitly regularizing the Jacobian of the encoder.

#### 5.1.3. Generic Applicability

**Figure 7:**
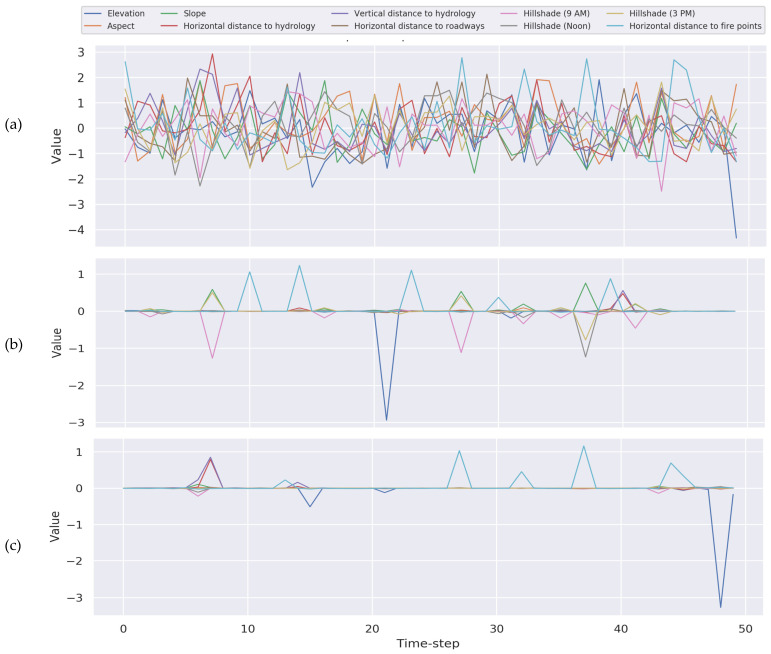
Auto-encoder training with different base models: (**a**) raw signal, (**b**) TSInsight attribution for the CNN and (**c**) TSInsight attribution for LSTM.

TSInsight is compatible with any base model. We tested our method with two prominent architectural choices in time series data, i.e., convolutional neural network (CNN) and LSTM. The results highlight that TSInsight was capable of extracting the salient regions of the input regardless of the underlying architecture. It is interesting to note that since LSTM uses memory cells to remember past states, the last point was found to be the most salient. For the CNN on the other hand, the network had access to the complete information, resulting in equal distribution of the saliency. A visual example is presented in Figure 7.

### 5.2. Loss Landscape

**Figure 8:**
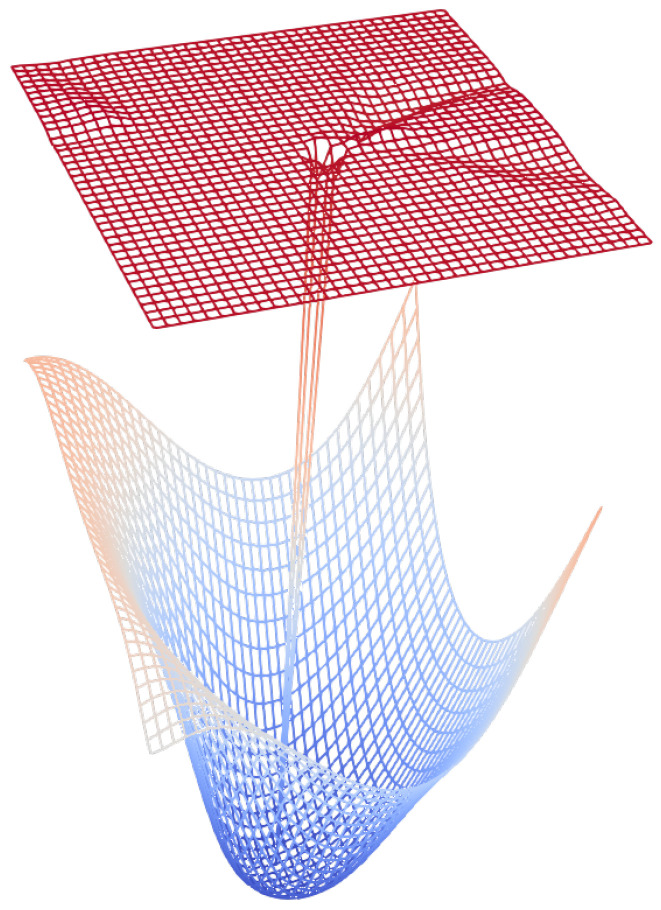
Loss landscape where the bottom surface indicates the manifold for the classifier while the surface on the top indicates the manifold for the auto-encoder attached to the classifier.

We analyzed the loss landscape in order to asses the impact of stacking the auto-encoder on top of the original network on the overall optimization problem. We followed the scheme suggested by Li et al. (2018) [36] where we first performed filter normalization using the norm of the filters. This allows the network to be scale invariant. We then sampled two random directions (δ and η) and used a linear combination of these directions to identify the loss landscape. We kept the values of the classifier in the combined model intact since we treat those parameters as fixed. The function representing the manifold can be written as:

(10) $$\begin{array}{c} f\left(\alpha ,\beta \right)=L\left(\theta^{*}+\alpha \delta +\beta \eta \right)\;\forall \alpha ,\beta \in \left\{-1.0,-0.95,-0.90,...,0.90,0.95,1.0\right\} \end{array}$$

where we iterate over different values of α and β from −1 to +1 with a fixed step size. Once the loss function is evaluated for all of the values of α and β (4000 different combinations), we plot the resulting function as a 3D surface. This loss landscape for the model trained on the forest cover dataset is visualized in Figure 8. The surface at the bottom (mostly in blue) signifies the loss landscape for the classifier. The landscape is nearly convex around the local minima found during the optimization. The surface on the top is from the model coupled with the auto-encoder. It can be seen that the loss landscape has a kink at the optimal position but remains otherwise flat with a significantly higher loss value. This indicates that the problem of optimizing the auto-encoder using gradients from the classifier is a significantly harder one to solve. This is consistent with our observation where the network failed to converge in many cases. Similar observations have been made by Palacio et al. [11] where they failed to fine-tune the complete auto-encoder, resorting to only fine-tuning of the decoder to make the problem tractable. The results were very similar when tested on other datasets.

## 6. Conclusions

We presented a novel method to discover the salient features of the input for the prediction by using the global context. With the obtained results, it is evident that the features highlighted by TSInsight are intelligible as well as reliable at the same time. In addition to interpretability, TSInsight also offers off-the-shelf properties which are desirable in a wide range of problems. Interpretability is essential in many domains, and we believe that our method opens up a new research direction for the interpretability of deep models for time series analysis. One major limitation of the current approach is the difficulty in tuning the hyperparameters (γ and β) which offers a good compromise between the final accuracy of the classifier and the interpretability of the model. It is non-trivial to define a simple scoring measure since interpretability itself is a subjective attribute. Therefore, we would like to further investigate the automated selection of the hyperparameters β and γ. We would also like to extend our experiments to the case of regression. Regression is usually a much harder problem to tackle since it lacks a direct causal interpretation. Another very interesting direction is to analyze the impact on the vulnerability of the classifier to adversarial examples with this sparse representation since the model now exploits the subspace of input instead of the complete space. This reduction in the input space may reduce the effectiveness of adversarial attacks on the final classifier.

Shoaib Ahmed Siddiqui, Dominique Mercier, Andreas Dengel and Sheraz Ahmed

## Figures and Tables

**Table 1:** Dataset details.

Dataset	Train	Validation	Test	Seq. Length	Input Ch.	# Classes
Synthetic Anomaly Detection	45000	5000	10000	50	3	2
Electric Devices	6244	2682	7711	50	3	7
Character Trajectories	1383	606	869	206	3	20
FordA	2520	1081	1320	500	1	2
Forest Cover	107110	45906	65580	50	10	2
ECG Thorax	1244	556	1965	750	1	42
WESAD	5929	846	1697	700	8	3
UWave Gesture	624	272	3582	946	1	8

**Table 2:** Hyperparameters.

Hyperparameter	Value
Initial learning rate	0.0001
Learning rate reduction factor	0.9
Learning rate reduction tolerance	4
Activation regularization (L1) - β	0.0001
Reconstruction weight - γ	4.0
Max epochs	50
Batch size	256
Early stopping patience	10

**Table 3:** Results for the different datasets in terms of accuracy for both the classifier as well as TSInsight.

Dataset	Model	γ	β	Accuracy	Difference
Synthetic Anomaly	Raw classifier	-	-	98.01 %	
Detection	TSInsight	1.0	0.001	98.13 %	+0.12 %
WESAD	Raw classifier	-	-	99.94 %	
	TSInsight	2.0	0.00001	99.76 %	−0.18 %
Character Trajectories	Raw classifier	-	-	97.01 %	
	TSInsight	0.25	0.0001	97.24 %	+0.23 %
FordA	Raw classifier	-	-	91.74 %	
	TSInsight	2.0	0.0001	93.26 %	+1.52 %
Forest Cover	Raw classifier	-	-	95.79 %	
	TSInsight	4.0	0.0001	96.26 %	+0.47 %
Electric Devices	Raw classifier	-	-	65.14 %	
	TSInsight	4.0	0.0001	65.74 %	+0.60 %
ECG Thorax	Raw classifier	-	-	86.01 %	
	TSInsight	0.1	0.0001	84.07 %	−1.94 %
UWave Gesture	Raw classifier	-	-	91.76 %	
	TSInsight	4.0	0.0005	92.29 %	+0.53 %

## Data Availability

Not applicable.
